# Diagnostic Accuracy of High‐Resolution Manometry for Assessing Fundoplication Integrity: Validation of the Padova Classification

**DOI:** 10.1111/nmo.70457

**Published:** 2026-10-02

**Authors:** Arianna Vittori, Giovanni Capovilla, Matteo Santangelo, Rena Yadlapati, C. Prakash Gyawali, Edoardo Vincenzo Savarino, Luca Provenzano, Francesca Forattini, Matteo Pittacolo, Irene Sole Zuin, Giulia Nezi, Loredana Nicoletti, Michele Valmasoni, Renato Salvador

**Affiliations:** ^1^ Department of Surgery, Oncology and Gastroenterology, 1st General Surgery Unit University of Padua, School of Medicine Padua Italy; ^2^ Department of Gastroenterology and Hepatology University of California San Diego San Diego California USA; ^3^ Division of Gastroenterology Washington University School of Medicine St Louis Missouri USA

**Keywords:** fundoplication, gastroesophageal reflux, manometry, surgery

## Abstract

**Background:**

Recurrent symptoms after laparoscopic fundoplication (LF) are not fully understood, with ongoing research focusing on their mechanisms. This study aimed to validate the Padova Classification, a postoperative high‐resolution manometry (HRM) framework to distinguish functioning from defective fundoplications.

**Methods:**

Prospectively collected data from consecutive patients who underwent post‐LF HRM (2010–2025) were analyzed and categorized as functioning and effective LF (FELF), post‐fundoplication outflow obstruction (PFOO), and ineffective neo‐barrier (INB). Primary analysis involved between‐group comparisons. Padova Classification diagnostic accuracy was evaluated against a composite reference standard (clinical, radiological, endoscopic, and pH‐monitoring criteria) by calculating sensitivity, specificity, positive predictive value, and negative predictive value for each diagnostic category. Diagnostic performance of postoperative integrated relaxation pressure (IRP) for identifying PFOO was evaluated using receiver operating characteristic curve analysis.

**Key Results:**

Among 373 patients (42.4% female; median age 54 years, median BMI 25.9 kg/m^2^; 55.2% Nissen, 44.8% Toupet), FELF, INB, and PFOO were observed in 58.7%, 21.4%, and 18.0% of patients, respectively, with PFOO most frequently seen with Nissen fundoplication (*p* = 0.001). The Padova Classification showed sensitivity/specificity 94.7%/97.1% for FELF, 88.8%/96.9% for INB, and 100%/97.4% for PFOO. Postoperative integrated relaxation pressure accurately discriminated PFOO (AUC 0.959–0.984; cutoff 18.1 mmHg; sensitivity 97.7%–98.0%, specificity 93.2%–96.8%), with no difference between ROC curves derived from Padova Classification and reference standard (*p* = 0.12).

**Conclusions:**

This study confirms that the Padova Classification demonstrates strong performance in assessing postoperative LF function.

## Key Points

- Persistent or recurrent symptoms after antireflux surgery can result from different underlying mechanisms that require different treatments.
- The Padova Classification provides a standardized way to interpret postoperative esophageal anatomical and physiological configurations and identify the cause of these symptoms.
- This study demonstrates that the Padova Classification can reliably characterize postoperative fundoplication function and may serve as a useful tool in the evaluation of patients after antireflux surgery.

## Plain Language Summary

Antireflux surgery is an effective treatment for gastroesophageal reflux disease, but some patients continue to experience symptoms such as heartburn, regurgitation, or difficulty swallowing after surgery. Determining whether these symptoms are caused by recurrent reflux, an overly tight surgical repair, or other postoperative changes can be challenging. The Padova Classification is a standardized system that uses esophageal pressure testing, known as high‐resolution manometry, to assess how well the surgical repair is functioning. In this study, we evaluated the Padova Classification in 373 patients who underwent laparoscopic fundoplication. The classification accurately distinguished between normal postoperative function, obstruction caused by an overly tight repair, and failure of the antireflux barrier. These findings provide a comprehensive clinical validation of the Padova Classification and suggest that it may serve as a practical tool for interpreting postoperative changes, helping clinicians identify the causes of persistent symptoms and guide further evaluation and treatment after antireflux surgery.

## Graphical abstract

The Padova Classification accurately characterized fundoplication function on high‐resolution manometry in 373 patients, showing high sensitivity (88.8%–100%) and specificity (96.9%–97.4%) against a composite reference standard, reliably distinguishing functioning, ineffective, and obstructive fundoplication phenotypes.

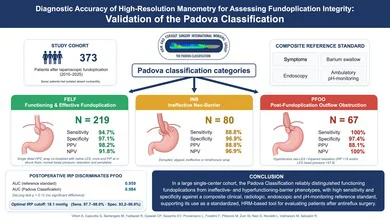

## 1 Introduction

Laparoscopic fundoplication (LF) is the surgical technique of choice for treating gastroesophageal reflux disease (GERD) [1, 2, 3]. The outcome after LF is reportedly good in approximately 85%–95% of patients at 5 years, while effective and sustained functional outcome is described in 80% of patients in the few studies with longer follow‐up [4, 5, 6]. Nevertheless, a subset of patients experiences persistent or recurrent symptoms following LF. Various mechanisms have been proposed, including a hyperfunctional or ineffective neo‐barrier, which could reduce the effectiveness of surgery and patients' willingness to undergo surgical treatment [7]. Since the reasons for a suboptimal outcome are not evident from symptoms alone, understanding the mechanisms underlying postoperative dysfunction and identifying predictors of poor outcomes after LF remain an active area of research in order to identify appropriate management strategies.

Postoperative assessment after LF can be challenging. While endoscopy and barium swallow studies may identify anatomical abnormalities, esophageal physiologic testing provides critical functional information that can help elucidate the causes of recurrent symptoms and guide clinical decision‐making [8]. In the absence of objective evidence of structural postoperative abnormalities, high‐resolution manometry (HRM) may be useful for the assessment and characterization of post‐fundoplication patients [9, 10]. Recently, an international multidisciplinary working group developed the Padova Classification to define an HRM‐based manometric profile and to determine whether HRM can reliably distinguish a defective or overly tight fundoplication from a well‐functioning wrap [11].

The aim of this study was to test and validate the newly proposed postoperative HRM‐based classification, the Padova Classification, and to verify its ability to systematically characterize esophagogastric junction (EGJ) function after LF using composite criteria, based on clinical evaluation, endoscopy, radiology, and reflux monitoring as the reference standard.

## 2 Material and Methods

### 2.1 Patient Population

We retrospectively analyzed prospectively collected data from January 2010 to June 2025 on all patients who underwent post‐fundoplication HRM at our institution, between 6 to 12 months after surgery, irrespective of the presence or absence of symptoms. The study was conducted at a teaching hospital and high‐volume referral center for esophageal diseases in Italy.

Patients who underwent postoperative assessment with conventional manometry had missing essential manometric data, lacked an available manometric report, or did not undergo at least one of the examinations required to establish the reference standard criteria for each category of the Padova Classification were excluded from the analysis. This study, based on data collected during routine clinical care without additional interventions performed for research purposes, was conducted in accordance with the ethical principles of the Declaration of Helsinki.

This study was conducted in accordance with the Standards for Reporting Diagnostic Accuracy Studies (STARD) guidelines and the Strengthening the Reporting of Observational Studies in Epidemiology (STROBE) statement [12, 13]. Completed checklists are provided in the Tables S1 and S2.

For patients who underwent surgery at our institution, the following preoperative and postoperative follow‐up protocols were adopted.

### 2.2 Preoperative Work‐Up

Patients' clinical characteristics were recorded. GERD symptoms were evaluated using the gastroesophageal reflux disease questionnaire (GERDQ) [14]. Preoperative dysphagia was assessed separately by combining severity (0 = none, 2 = mild, 4 = moderate, 6 = severe) with frequency (0 = never, 1 = occasionally, 2 = once a month, 3 = weekly, 4 = twice a week, 5 = daily) [15, 16]. All patients underwent preoperative evaluation with esophagogastroduodenoscopy (EGD), barium swallow, esophageal manometry (high‐resolution or conventional), and 24‐h pH‐impedance or 96‐h wireless pH monitoring. A conclusive diagnosis of GERD was established according to the guidelines available at the time of surgery [17, 18, 19].

### 2.3 Postoperative Assessment

All patients were evaluated after LF using the GERDQ. Dysphagia was assessed separately using the same approach adopted in the preoperative evaluation. Patients also underwent barium swallow (BS), EGD, HRM, and 24‐h pH‐impedance.

A barium swallow study was routinely performed approximately 1 month postoperatively according to our institutional imaging protocol based on the timed barium swallow technique described by de Oliveira et al. [20] All studies were blindly reviewed by an experienced gastrointestinal radiologist with dedicated expertise in postoperative antireflux surgery imaging to assess the morphology and position of the fundoplication, esophageal and EGJ transit, and the presence of recurrent hiatal hernia [21, 22].

The HRM protocol has been described elsewhere [23, 24]. The procedure was performed between 6 to 12 months from surgery using a catheter 4.2 mm in diameter with 36 solid‐state circumferential sensors spaced at 1‐cm intervals and spanning the whole esophagus (Medtronic, Minneapolis, MN, USA). Ambulatory 24‐h pH‐impedance monitoring was performed off therapy in accordance with established protocols to evaluate esophageal acid exposure.

Detailed endoscopic assessment of the fundoplication was performed within 1 year from surgery, as per our institutional protocol, according to the criteria proposed by Mittal et al. [25] Endoscopic examinations were performed by experienced esophageal surgeons familiar with the standardized postoperative assessment of fundoplication. For each procedure, the following parameters were recorded: the location of the EGJ relative to the neo‐valve and the crura, the competence of the wrap, and its spatial orientation in relation to the diaphragmatic crura. The endoscopic findings were categorized as follows: (a) well‐represented fundoplication, (b) too‐tight fundoplication, (c) intrathoracic fundoplication, (d) slipped fundoplication, and (e) disrupted fundoplication. Esophagitis was staged according to the Los Angeles classification [26]. Any area of erythema or velvety‐appearing mucosa in the esophagus was considered suspicious for non‐native epithelium at endoscopy. A diagnosis of Barrett's esophagus (BE) was established only after histopathological confirmation with hematoxylin and eosin staining demonstrated intestinal metaplasia [27].

### 2.4 Application of the Padova Classification

Postoperative HRM studies were interpreted according to the Padova Classification. The classification was applied using a stepwise approach based first on postoperative EGJ anatomy, followed by neo‐lower esophageal sphincter (neo‐LES) physiology.

The initial assessment determined whether there was > 1 cm separation between the neo‐LES and the crural diaphragm (CD). When a double high‐pressure zone (HPZ) was present and the distal HPZ was located below the CD, the study was classified as slipped fundoplication. When a double‐HPZ configuration was present and the neo‐LES/fundoplication complex was located above the CD, basal neo‐LES pressure was used to distinguish intrathoracic fundoplication from disrupted fundoplication with herniation; normotensive basal pressure was classified as intrathoracic fundoplication, whereas hypotensive LES (i.e., LES basal pressure < 11.2 mmHg for Nissen fundoplication and 8.4 mmHg for Toupet fundoplication) [9] was classified as disrupted fundoplication with herniation.

In cases without separation between the neo‐LES and the CD, classification was based on functional findings. A hypotensive basal neo‐LES was classified as disrupted or ineffective fundoplication. A hypertensive basal neo‐LES and/or impaired neo‐LES relaxation, reflected by IRP > 19 mmHg and/or LES basal pressure > 47.8 mmHg, was classified as post‐fundoplication outflow obstruction (PFOO); the presence of intrabolus pressurization was considered supportive of this diagnosis. If basal pressure and relaxation were not abnormal, esophageal peristalsis was then assessed. Absent contractility was defined as failed peristalsis in 100% of swallows. When none of the above abnormalities were present, the HRM pattern was considered consistent with a functioning and effective LF (FELF), characterized by a single distal esophageal HPZ with colocalization of the wrap and native LES, with crura and pressure inversion point (PIP) at or above them. The definitions of each Padova Classification category are summarized in Table 1. All HRM reports were reviewed by three experts (RS, GC, LP) blinded to patients' follow‐up procedures and surgical outcomes. Although interobserver agreement was not formally quantified, final classification was reached by consensus.

**TABLE 1:** Summary of postoperative high‐resolution manometry category definitions based on the Padova Classification.

Padova category	Definition
Functioning and effective laparoscopic fundoplication (FELF)	Single distal esophageal high‐pressure zone (HPZ) with colocalization of the wrap and native lower esophageal sphincter (LES); crura and pressure inversion point (PIP) at or above them; normal basal pressure and relaxation; normal peristalsis.
Intrathoracic fundoplication	Double HPZ present; neo‐LES located above the crural diaphragm (CD); normotensive basal LES pressure (≥ 11.2 mmHg for Nissen, ≥ 8.4 mmHg for Toupet).
Disrupted fundoplication with herniation	Double HPZ present; neo‐LES located above CD; hypotensive basal LES pressure (< 11.2 mmHg for Nissen, < 8.4 mmHg for Toupet).
Slipped fundoplication	Double HPZ present; distal HPZ located below the CD.
Disrupted or ineffective fundoplication	No separation between neo‐LES and CD; hypotensive basal neo‐LES (< 11.2 mmHg for Nissen, < 8.4 mmHg for Toupet).
Post‐fundoplication outflow obstruction (PFOO)	No separation between neo‐LES and CD; hypertensive basal neo‐LES and/or impaired relaxation (IRP > 19 mmHg and/or LES basal pressure > 47.8 mmHg); intrabolus pressurization supportive.
Absent contractility	Failed peristalsis in 100% of swallows.

The reported manometric pressure values apply to Medtronic systems.

### 2.5 Reference Standard Criteria for Post‐Fundoplication Evaluation

Diagnostic accuracy of the Padova Classification was assessed against a composite reference standard based on clinical, radiologic, endoscopic, and ambulatory pH‐monitoring findings. The reference standard was chosen according to previously published radiological and surgical classifications, reflecting expert clinical consensus and current practice [7, 9, 16, 19, 22, 28, 29, 30].

PFOO diagnosis required the presence of dysphagia (dysphagia intensity scores > 2) [9] together with a barium swallow study showing delayed esophageal emptying, defined as persistent retention of a contrast column measuring > 5 cm above the esophagogastric junction at 1 min [28, 29]. Disrupted or ineffective fundoplication was defined by abnormal ambulatory pH monitoring (acid exposure time [AET] > 6%) [19] with or without endoscopic evidence of esophagitis of Los Angeles (LA) grade B or higher. Intrathoracic fundoplication diagnosis involved the presence of an abnormal barium swallow, with or without abnormal pH monitoring and/or indicative endoscopic findings [7, 22]. Disrupted fundoplication with herniation was defined by a suggestive barium swallow together with abnormal pH monitoring and/or LA grade B or higher esophagitis. Slipped fundoplication was detected by a characteristic barium swallow, with abnormal pH monitoring, and/or suggestive endoscopic findings [22, 31]. Finally, FELF was defined by a normal barium swallow, normal pH monitoring, and normal endoscopy or esophagitis of LA grade A. A summary of the reference standard criteria is shown in Figure 1. When one component of the reference standard was unavailable, classification was based on the remaining available tests, provided sufficient information was present to establish postoperative functional status. Assessment of the composite reference standard was performed by three experts (RS, GC, LP) without knowledge of HRM findings or Padova Classification results.

**FIGURE 1:**
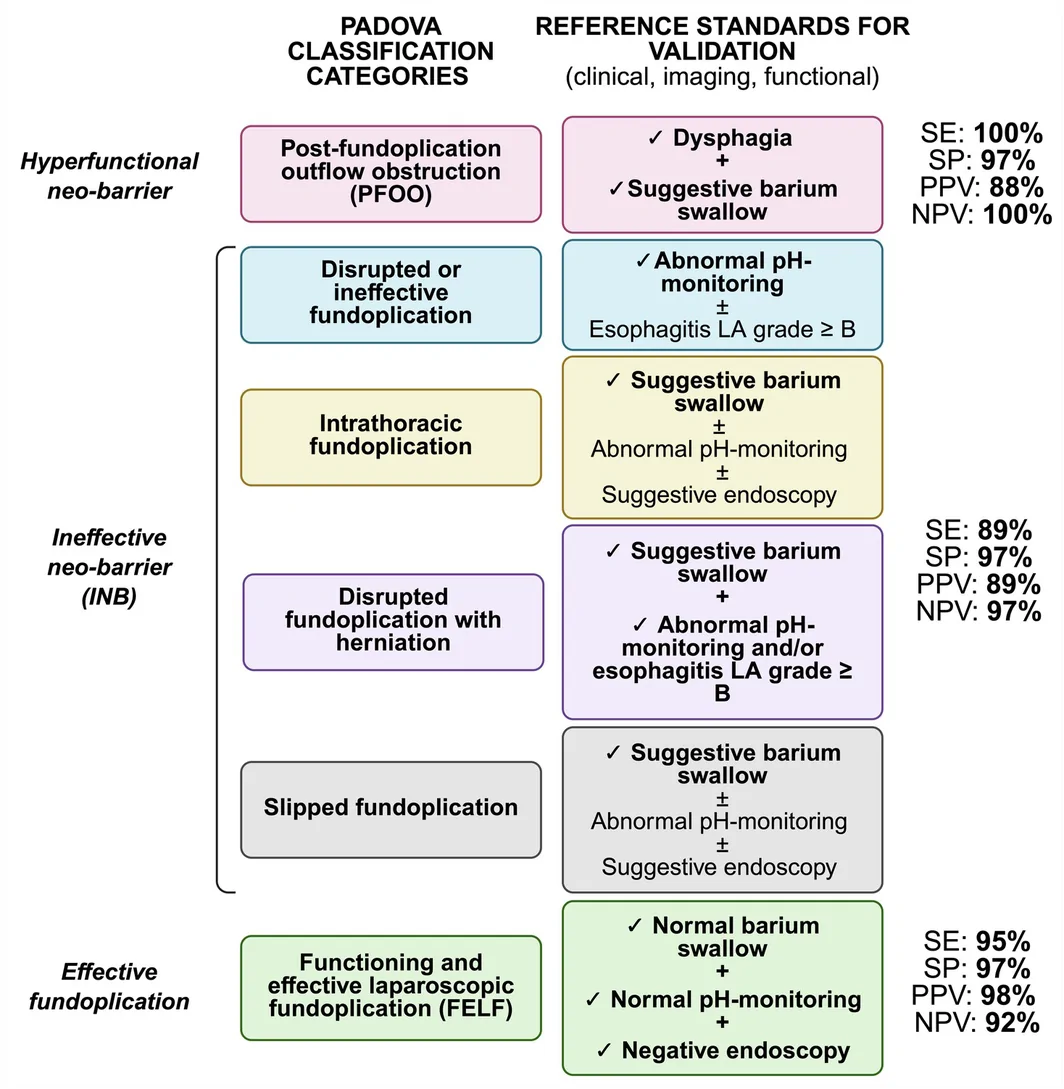
Diagnostic framework for post‐fundoplication evaluation. High‐resolution manometry patterns according to the Padova Classification are shown together with their corresponding reference diagnostic standard used for validation, based on symptoms, imaging findings, and functional tests. The diagnostic accuracy of the three Padova HRM categories—hyperfunctional neo‐barrier, ineffective neo‐barrier, effective and functioning laparoscopic fundoplication—is reported on the right. Mandatory criteria are indicated by a checkmark (✓). AET, acid exposure time; LA, los angeles classification; NPV, negative predictive value; SE, sensitivity; SP, specificity; PPV, positive predictive value. Created in https://BioRender.com (University of Padova license).

For statistical analysis, patients were grouped into three major categories: hyperfunctional neo‐barrier (i.e., PFOO), FELF, and ineffective neo‐barrier (INB), which includes disrupted or ineffective fundoplication, disrupted fundoplication with herniation, intrathoracic stomach, and slipped fundoplication. Because the individual anatomical subtypes comprising the ineffective neo‐barrier category occurred infrequently, diagnostic accuracy analyses were performed at the level of the three principal postoperative functional phenotypes (FELF, PFOO, and INB). Accordingly, the present study was designed to validate the Padova Classification as a functional diagnostic framework rather than to estimate the diagnostic performance of each anatomical subtype separately.

Patients classified as FELF according to the Padova Classification, who had normal radiologic, endoscopic, and pH‐monitoring findings but a GERDQ score > 8, were further evaluated using the Esophageal Hypervigilance and Anxiety Scale (EHAS) [32, 33]. The EHAS is a 15‐item self‐report questionnaire designed to evaluate esophageal hypervigilance and symptom‐specific anxiety over the past month. Each item is rated on a 5‐point Likert scale (0–4), and the total score is calculated by summing all items. The overall EHAS score ranges from 0 to 60, with higher values reflecting greater levels of esophageal hypervigilance and anxiety related to esophageal symptoms. A total score > 21 indicates clinically elevated symptoms.

No indeterminate index test or reference standard results were observed.

### 2.6 Statistical Analysis

A convenience sample was used, including all consecutive eligible patients identified during the study period. Patients with absent contractility at both preoperative and postoperative assessments were excluded from the statistical analysis. Continuous variables were assessed for normality using the Shapiro–Wilk test and visual inspection of histograms and Q‐Q plots. Data are presented as mean ± standard deviation for normally distributed variables and as median with interquartile range for non‐normally distributed variables. Statistical analyses were performed to compare demographic and clinical characteristics among the FELF, PFOO, and INB groups. Comparisons of continuous variables among the three groups were performed using the Kruskal–Wallis test. Comparisons between two independent groups were performed using the Student's *t*‐test for normally distributed variables and the Wilcoxon rank‐sum test for non‐normally distributed variables. Categorical variables are reported as frequencies and percentages and were compared using the chi‐square or Fisher's exact test, as appropriate. A two‐sided *p* value < 0.05 was considered statistically significant. The diagnostic performance of the Padova Classification was evaluated against the composite reference standard by calculating sensitivity, specificity, positive predictive value (PPV), and negative predictive value (NPV) for each diagnostic category. No subgroup analyses of variability in diagnostic accuracy were pre‐specified. The diagnostic performance of postoperative integrated relaxation pressure (IRP) for identifying PFOO was evaluated using receiver operating characteristic (ROC) curve analysis, with the reference standard diagnosis used as the classification benchmark. Area under the curve (AUC) values were calculated, and optimal cutoff values were determined using Youden's index. ROC analyses were further stratified by fundoplication type, and AUC differences were compared using DeLong's test. Statistical analyses were performed using R software (version 4.5.2; R Foundation for Statistical Computing, Vienna, Austria).

## 3 Results

A total of 373 patients were included in the analysis (158 [42.4%] female, median age 54 years [IQR, 43–64], and median body mass index 25.9 kg/m^2^ [IQR, 23.5–28.1]). The flowchart of the study population selection is presented in Figure S1. All procedures were completed laparoscopically. Fundoplication was performed according to the Nissen technique in 206 patients (55.2%) and the Toupet technique in 167 patients (44.8%). Based on the Padova Classification, 219 (58.7%) patients had a postoperative manometric diagnosis of FELF, 80 (21.4%) patients had an INB, and 67 (18%) patients had a PFOO. Baseline demographic and preoperative clinical characteristics in the three groups are summarized in Table S3. In the INB group, 47 (58.8%) patients had an intrathoracic fundoplication, 20 (25%) had a disrupted or ineffective fundoplication, 7 (8.8%) had a slipped fundoplication, and 6 (7.4%) had a disrupted fundoplication with herniation. Seven (1.9%) patients had absent contractility; therefore, they were excluded from further analysis. Four out of 219 (1.8%) patients diagnosed with FELF according to both the Padova Classification and the reference standard also had an abnormal GERDQ score. These patients subsequently underwent further symptom evaluation using the EHAS, which was > 21 in all cases.

### 3.1 Differences in Distribution of Preoperative Variables Across Padova Categories

The distribution of preoperative variables across Padova Classification categories is shown in Figure 2. Preoperative LES total length, LES abdominal length, LES basal pressure, and IRP differed significantly between groups, as shown in Table S3. However, post hoc comparisons demonstrated significant differences only for LES abdominal length (*p* = 0.004) and LES total length (*p* = 0.001) between the PFOO and the other categories. Preoperative AET differed significantly between groups (Kruskal–Wallis *χ* ^2^ = 13.63, df = 2, *p* < 0.001). Wilcoxon rank‐sum testing confirmed the difference between the INB group and the other categories (*p* < 0.001, Figure 3).

**FIGURE 2:**
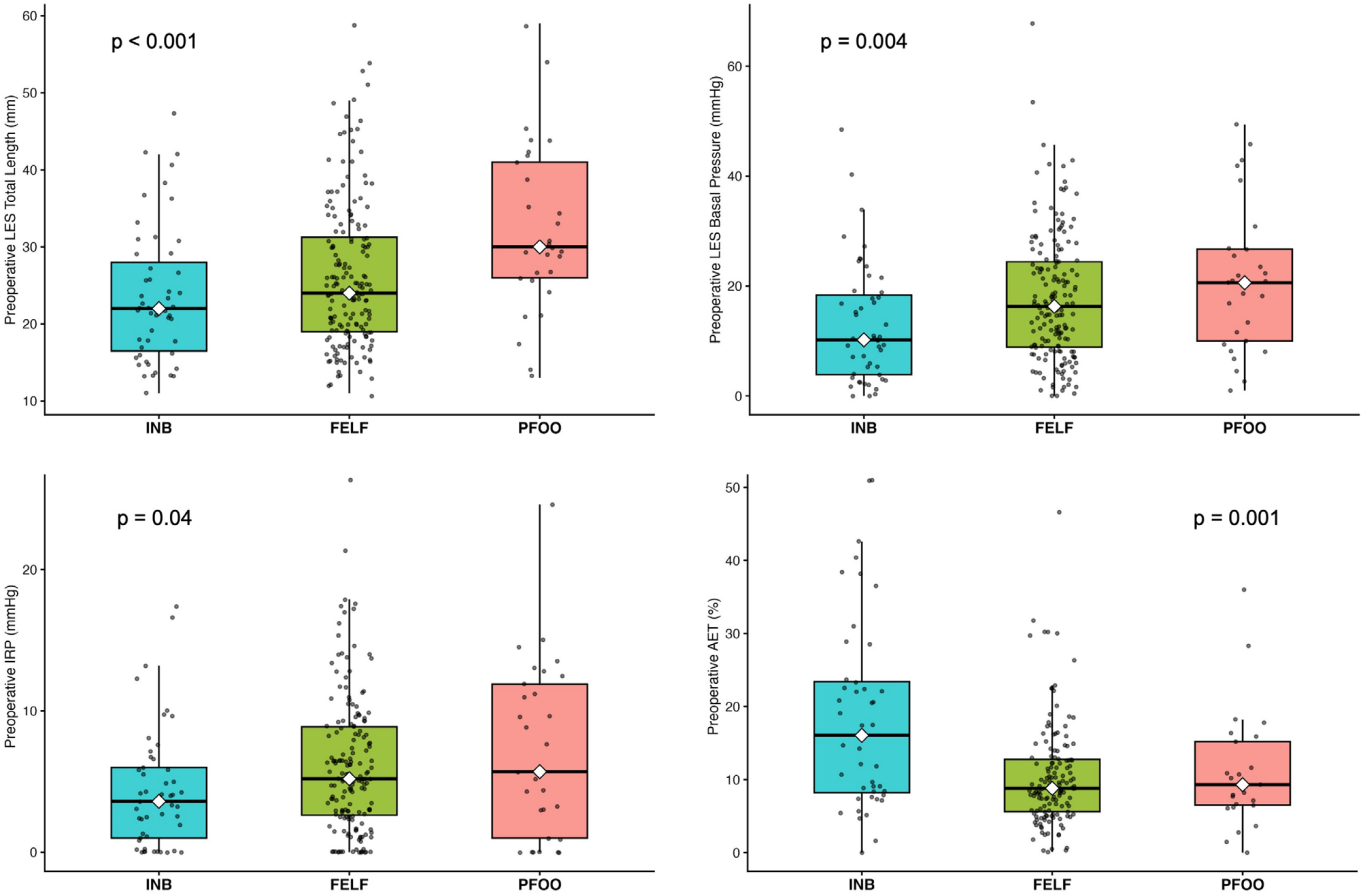
Distribution of preoperative variables across Padova Classification categories. AET, acid exposure time; FELF, functioning and effective fundoplication; INB, ineffective neo‐barrier; IRP, integrated relaxation pressure; LES, lower esophageal sphincter; PFOO, post‐fundoplication outflow obstruction.

**FIGURE 3:**
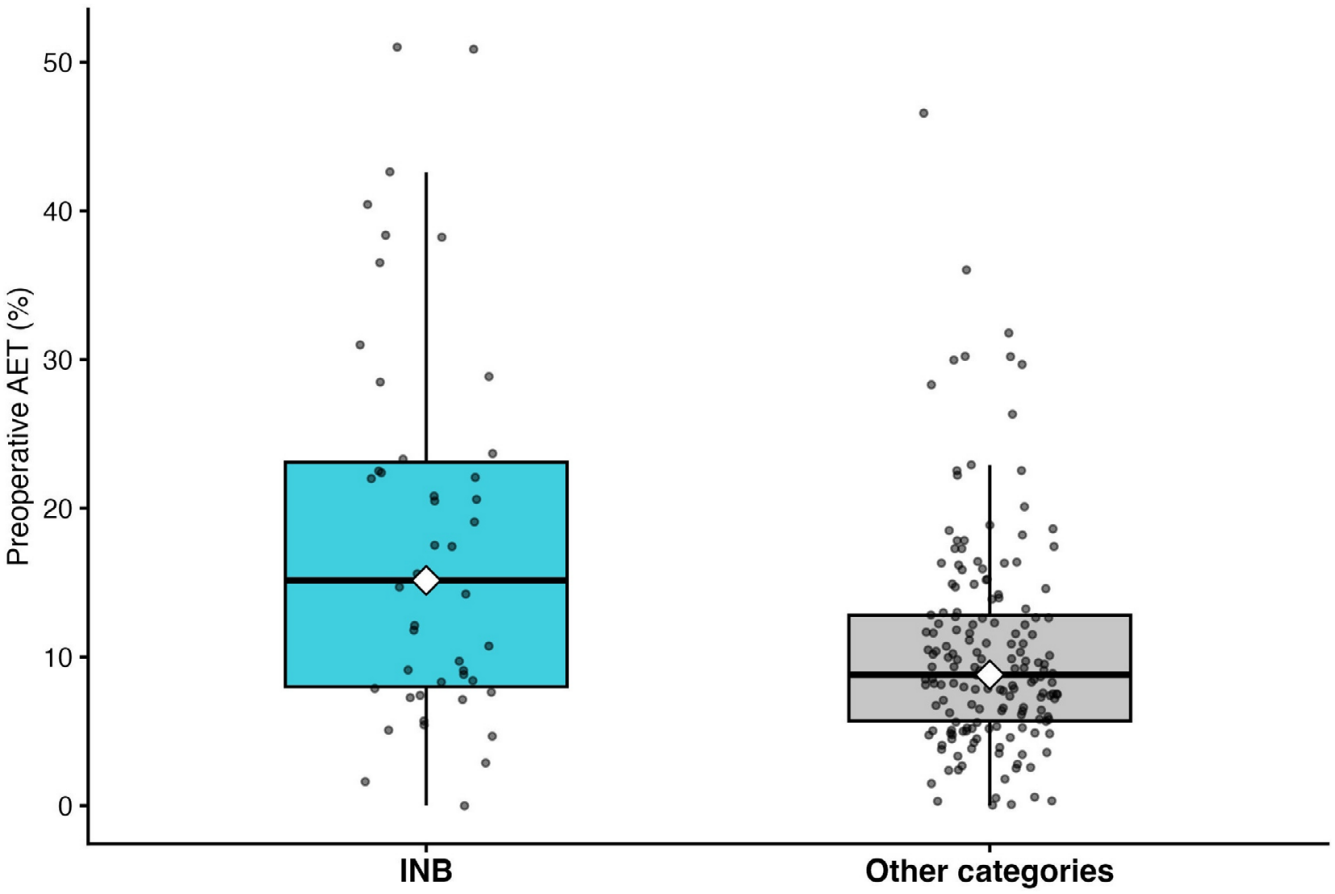
Distribution of preoperative acid exposure time (AET) in patients classified as ineffective neo‐barrier (INB) compared with the remaining Padova categories. Differences between groups were assessed using the Wilcoxon rank‐sum test (*p* < 0.001).

The distribution of fundoplication types differed significantly across Padova Classification groups (Pearson's chi‐square test with Yates' continuity correction, *χ* ^2^ = 18.70, *p* < 0.001). Similarly, comparison of PFOO versus other categories demonstrated a significant association between PFOO and fundoplication types (*χ* ^2^ = 11.49, *p* = 0.001). Notably, Nissen fundoplication was more prevalent among patients in the PFOO group compared with other categories. Effect size analysis showed a small‐to‐moderate association between fundoplication type and Padova Classification groups (Cramer's *V* = 0.229), indicating that although the distribution differed significantly, the magnitude of the association was limited.

### 3.2 Postoperative Characteristics

Postoperative manometric and pH‐impedance parameters across groups are summarized in Table 2. In the PFOO group, the median postoperative IRP was 19.8 mmHg (IQR, 19.0–22.0), compared with 9.7 mmHg (IQR, 7.0–12.3) in the FELF group and 8.7 mmHg (IQR 4.2–12.6) in the INB group. Postoperative IRP differed significantly across Padova Classification categories (Kruskal–Wallis *χ* ^2^ = 136.05, df = 2, *p* < 0.001), with a significant difference between the PFOO group and the other categories (*p* < 0.001). AET after surgery differed significantly among the three groups (Kruskal–Wallis *χ* ^2^ = 86.42, df = 2, *p* < 0.001), with values being higher in the INB group (median 6.2%, IQR, 0.9–10.9) compared with the FELF group (median 0.1%, IQR, 0.0–0.4) and the PFOO group (median 0.2%, IQR 0.2–1.0) (*p* < 0.001).

**TABLE 2:** Postoperative clinical characteristics in the three groups: functioning and effective laparoscopic fundoplication (FELF), ineffective neo‐barrier (INB), and post‐fundoplication outflow obstruction (PFOO).

Characteristic	FELF *N* = 219^a^	INB *N* = 80^a^	PFOO *N* = 67^a^	*p* ^b^
GERDQ score	2 (0–4)	6 (2–12)	2 (0–3)	< 0.001
LES total length, mm	32 (25–40)	25 (20–30)	39 (32–47)	< 0.001
LES abdominal length, mm	18 (10–24)	0 (0–12)	20 (16–28)	< 0.001
LES basal pressure, mmHg	21 (16–27)	16 (10–22)	36 (29–46)	< 0.001
IRP, mmHg	9.6 (7.0–12.3)	8.7 (4.2–12.6)	19.8 (19.0–22.0)	< 0.001
DCI, mmHg·s·cm	563 (340–745)	531 (256–735)	674 (327–833)	0.3
AET, %	0.1 (0.0–0.4)	6.2 (0.9–10.9)	0.2 (0.0–1.0)	< 0.001

Abbreviations: AET, acid exposure time; DCI, distal contractile integral; GERDQ, gastroesophageal reflux disease questionnaire; IRP, integrated relaxation pressure; LES, lower esophageal sphincter.

^a^ Median (Q1–Q3).

^b^ Kruskal–Wallis rank sum test.

### 3.3 Diagnostic Performance of the Padova Classification

Using the reference standards shown in Figure 1, the Padova Classification demonstrated high diagnostic performance across all three categories. The availability of each component of the composite reference standard, overall and according to the three Padova Classification diagnostic categories, is reported in Table S4. For FELF, sensitivity was 94.7% (95% confidence interval [CI], 90.9–97.2) and specificity was 97.1% (95% CI, 92.8–99.2), with a PPV of 98.2% (95% CI, 95.4–99.5) and a NPV of 91.8% (95% CI, 86.2–95.7). For INB, sensitivity was 88.8% (95% CI, 79.7–94.7) and specificity was 96.9% (95% CI, 94.1–98.6), with a PPV of 88.8% (95% CI, 79.7–94.7) and an NPV of 96.9% (95% CI, 94.1–98.6). Diagnostic performance was highest for PFOO, with a sensitivity of 100% (95% CI, 93.9–100) and a specificity of 97.4% (95% CI, 94.9–98.9); PPV was 88.1% (95% CI, 77.8–94.7) and NPV was 100% (95% CI, 98.8–100). The cross‐tabulation of index test and reference standard results for each investigated Padova Classification category is shown in Table S5.

ROC curve analysis was performed to evaluate the diagnostic performance of postoperative IRP for identifying PFOO in the Nissen group, using both the reference standard and the Padova Classification definitions of PFOO. Postoperative IRP demonstrated excellent discriminative ability in both analyses. When using the reference standard, the AUC was 0.959 (95% CI, 0.933–0.985), while using the Padova Classification the AUC was 0.984 (95% CI, 0.968–0.999) (Figure 4). The difference between the two ROC curves was not statistically significant (DeLong test *p* = 0.12). The optimal postoperative IRP cutoff for predicting PFOO was 18.1 mmHg in both models. For the reference standard, this threshold yielded a sensitivity of 97.7% and specificity of 93.2%, while for the Padova Classification sensitivity and specificity were 98.0% and 96.8%, respectively.

**FIGURE 4:**
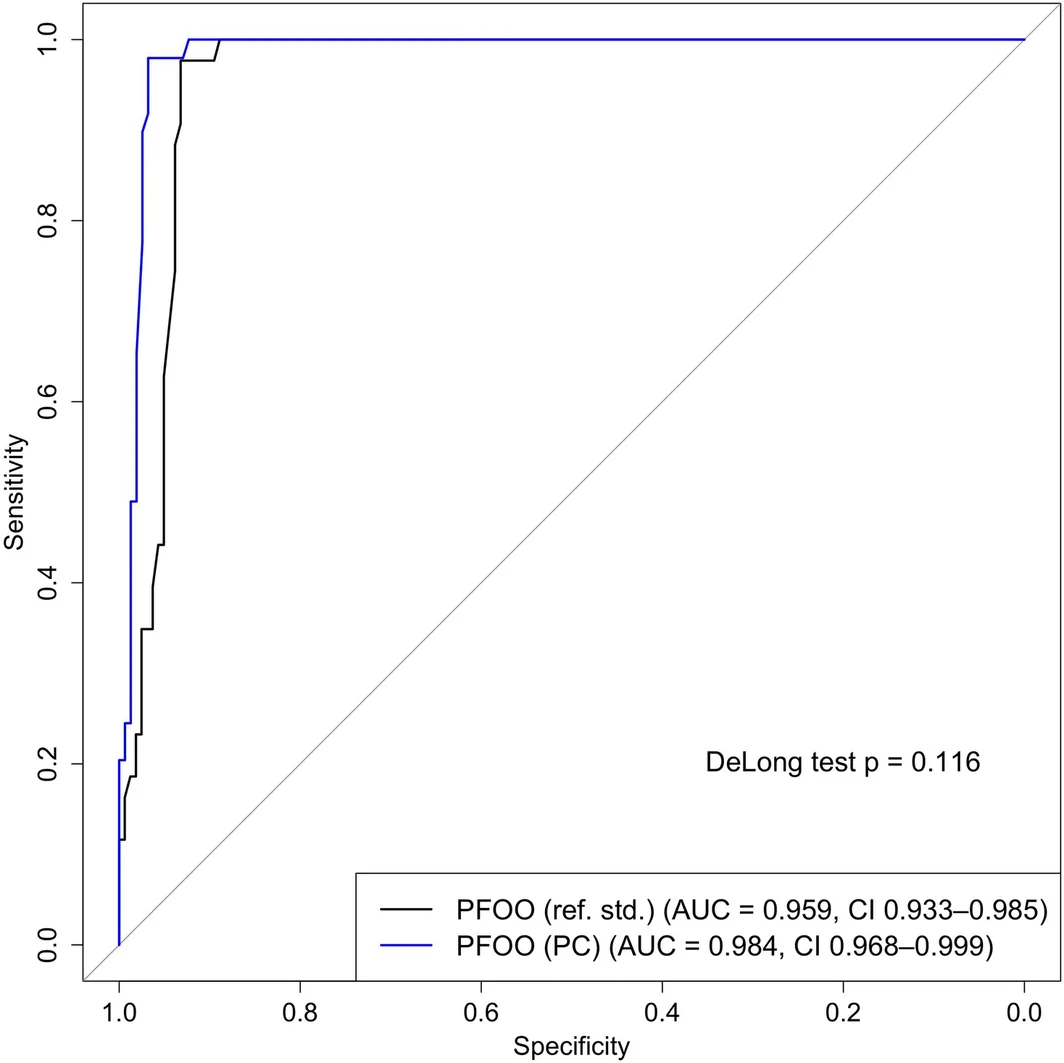
Receiver operating characteristic (ROC) curves evaluating the ability of postoperative integrated relaxation pressure (IRP) to discriminate post‐fundoplication outflow obstruction (PFOO) in patients undergoing Nissen fundoplication. ROC curves were generated using IRP as the continuous test variable and PFOO status as the binary outcome, defined according to two alternative definitions: (1) the composite reference standard (ref. std.) used for the Padova classification validation, generating the black curve; and (2) the Padova Classification (PC), generating the blue curve. The diagnostic performance of IRP was quantified by the area under the curve (AUC). The two ROC curves were statistically compared using the DeLong test. CI, confidence interval.

## 4 Discussion

This study sought to evaluate whether the Padova Classification can reliably differentiate between well‐functioning fundoplication wraps and those associated with mechanical dysfunction or abnormal esophageal outflow, and to determine its clinical utility in the assessment of patients with suspected post‐fundoplication physiological or anatomical abnormalities. In this large cohort of patients systematically evaluated with multimodal postoperative testing, the Padova Classification demonstrated high diagnostic accuracy in identifying the main functional phenotypes of fundoplication, including post‐fundoplication outflow obstruction, ineffective neo‐barrier configurations, and functioning and effective laparoscopic fundoplication. This represents the first clinical validation of the Padova Classification for the interpretation of postoperative HRM after LF.

Previous studies have explored the potential role of HRM in the evaluation of persistent and recurrent symptoms after fundoplication, particularly in patients with suspected ineffective antireflux barrier [34, 35, 36, 37]. In one of the earliest reports, Tatum and colleagues observed that a dual HPZ, reflecting spatial separation between the intrinsic LES and the CD, was significantly more frequent in patients with suboptimal surgical outcomes (57% vs. 9%) [34]. Abnormal acid exposure was observed in more than three‐quarters of patients with a dual HPZ compared with about one‐third of those with a single HPZ, suggesting that this manometric pattern reflects disruption of the antireflux barrier. Similarly, Hoshino et al. evaluated HRM pressure topography patterns in symptomatic post‐fundoplication patients and demonstrated that specific manometric configurations correspond to distinct anatomical alterations of the wrap identified endoscopically [35]. Intrathoracic and slipped fundoplication were frequently associated with a double HPZ, whereas disrupted fundoplication was characterized by reduced basal LES pressure. These findings supported the concept that HRM can help identify the mechanisms of ineffective antireflux barriers following fundoplication, particularly when a suboptimal outcome is related to anatomical migration or wrap disruption.

In a more recent study from our group, this concept was extended by moving from the descriptive recognition of abnormal postoperative patterns to the identification of the manometric profile of a functioning and effective fundoplication [9]. In a cohort of GERD patients studied before and after LF, we showed that patients with pathological postoperative acid exposure had a significantly shorter abdominal LES length after surgery and a higher prevalence of abnormal hiatal morphology, particularly intrathoracic fundoplication. At multivariable analysis, postoperative abdominal LES length and separation of the LES‐wrap complex above the diaphragm were independently associated with surgical failure, with two high‐pressure zones showing the strongest association among manometric parameters. Although these early investigations highlighted the potential of HRM in the postoperative setting, a standardized interpretive framework was still lacking.

To address this gap, an international multidisciplinary working group developed the Padova Consensus, introducing an algorithm‐based approach to postoperative HRM interpretation [11]. Building on the pathophysiological observations of prior studies, the classification integrates EGJ morphology and function into a stepwise diagnostic algorithm: the first step evaluates the spatial relationship between the neo‐LES and the CD, translating the dual HPZ pattern into defined anatomical categories, slipped fundoplication, intrathoracic migration, or herniation with disruption. In the absence of anatomical separation, classification is based on functional parameters, with a reduced basal neo‐LES pressure identifying a disrupted or insufficient wrap.

The present study validates the ability of the Padova Classification to characterize ineffective neo‐barrier configurations in a large, prospectively collected cohort. For the INB category, the classification demonstrated a sensitivity of 88.8% and a specificity of 96.9%, confirming its reliability in identifying patients with structural defects of the antireflux barrier. Postoperative manometric and pH‐impedance data further support the validity of this category: patients classified as INB exhibited significantly higher postoperative AET compared with both the FELF group and the PFOO group, alongside lower postoperative LES basal pressure and shorter abdominal LES length, consistent with loss of the functional antireflux barrier. Recognizing INB carries direct clinical implications: patients classified as INB present with objective failure of the antireflux barrier, reflected by significantly higher postoperative acid exposure, and may therefore require further anatomical evaluation to guide clinical management, in contrast to those with FELF in whom conservative or medical management may be appropriate.

The diagnostic performance of the Padova Classification was highest for PFOO. This finding is particularly relevant in light of the conceptual framework established by the Padova Consensus, which identified PFOO as a distinct manometric entity defined by a hypertensive or poorly relaxing neo‐LES, with elevated intrabolus pressure serving as a supportive criterion. The first validation of the PFOO criteria was provided by Forattini et al. in a multicenter international study, which demonstrated that patients with manometric PFOO exhibited significantly higher median LES basal pressure and IRP compared with those with FELF, and that dysphagia was present in 89% of PFOO patients versus only 5% of those with FELF [16]. The present study further validates the PFOO criteria in a larger cohort and extends this validation through ROC curve analysis of postoperative IRP against both the Padova Classification and an independent composite reference standard. Although the near‐perfect diagnostic performance of IRP against the Padova Classification may be expected, as elevated IRP constitutes one of the defining criteria for PFOO within the classification, this analysis was not intended to validate IRP against itself. Rather, the primary objective was to determine whether the ROC curve obtained using the independent composite reference standard was comparable to that obtained using the Padova Classification. The finding that both ROC curves showed excellent discrimination and identified the same optimal postoperative IRP cutoff indicates that the Padova Classification identifies essentially the same pathophysiological entity recognized through combined clinical, radiological, endoscopic, and pH‐monitoring assessment. This close agreement supports the clinical validity of the Padova framework and suggests that HRM‐based classification reliably reflects the postoperative phenotype identified by the multidisciplinary reference standard.

Interestingly, postoperative functional phenotypes were associated with distinct preoperative physiological characteristics. Patients who developed INB had greater baseline acid exposure and a shorter LES, with a lower basal pressure, suggesting more advanced disruption of the EGJ. This is physiologically plausible, as severe reflux disease is often accompanied by hiatal hernia, LES hypotonia, and altered EGJ anatomy, factors that may compromise the long‐term integrity of the fundoplication and increase the risk of recurrent reflux. This is consistent with prior reports [38, 39, 40] showing that patients with severe reflux disease, particularly those with large hiatal hernias or markedly abnormal acid exposure, are at increased risk of recurrent reflux after surgery due to the complexity of EGJ reconstruction and the potential for wrap disruption or migration. In contrast, patients who developed PFOO exhibited higher preoperative LES basal pressure, LES total length, and IRP, consistent with the hypothesis that a stronger native sphincter may contribute to a more restrictive neo‐EGJ after fundoplication. Although IRP is the primary metric for diagnosing EGJ outflow obstruction in the Chicago Classification, its ability to predict postoperative obstruction has been inconsistent, with several studies showing limited predictive value of baseline HRM metrics for postoperative dysphagia or functional obstruction after fundoplication [41, 42, 43]. Although this study was not designed to identify predictive factors, these observations suggest that preoperative esophageal physiology may influence postoperative functional outcomes and warrant further investigation to determine their prognostic value and potential role in preoperative risk stratification and counseling.

Several limitations of this study should be acknowledged. First, its single‐center design, although conducted at a high‐volume referral center with extensive expertise in esophageal surgery and HRM, may limit the generalizability of the findings. In addition, the absence of an independent external validation cohort and the involvement of some authors in the development of the Padova Classification may have introduced bias. Second, the composite reference standard relied on multiple complementary diagnostic modalities rather than a single gold standard. Although this approach reflects the multifactorial anatomical and physiological changes that characterize the post‐fundoplication EGJ, the included investigations were not always performed simultaneously. Nevertheless, all assessments were completed within the first postoperative year, making substantial bias from late anatomical changes unlikely while reflecting routine clinical practice. Furthermore, endoscopic examinations were performed by different operators and, despite the use of standardized protocols and published diagnostic criteria, retrospective review cannot completely eliminate interobserver variability. Third, the relatively small number of patients within individual INB subgroups necessitated their aggregation for analysis, precluding anatomical subgroup‐specific validation. Likewise, the limited number of patients with PFOO after Toupet fundoplication prevented subgroup ROC analyses. Moreover, the choice of fundoplication was not standardized but reflected surgeon preference based on GERD severity and preoperative esophageal motility, with a progressive decline in the use of Nissen fundoplication during the study period, potentially introducing selection and temporal bias. Fourth, although HRM studies and the composite reference standard were reviewed independently in separate sessions with reciprocal blinding, the use of the same expert panel for both assessments may have introduced recall bias. Finally, esophageal distensibility was not assessed using the functional lumen imaging probe (FLIP), an emerging complementary technique for evaluating EGJ function after fundoplication. In addition, although the Padova Classification demonstrated excellent diagnostic performance in this study, its impact on clinical decision‐making and patient outcomes remains to be established.

These limitations point to several priorities for future research. Larger cohorts and prospective studies across diverse clinical settings are needed to confirm the generalizability of the Padova Classification and support its broader adoption. Finally, integration with complementary modalities, particularly FLIP, may further refine diagnostic accuracy and deepen our understanding of post‐fundoplication physiology.

## 5 Conclusion

In conclusion, this study confirms that the Padova Classification demonstrates strong performance in assessing postoperative LF function and may serve as a valuable tool in clinical decision‐making.

## Author Contributions

Conceptualization: Arianna Vittori, Giovanni Capovilla, Matteo Santangelo, Michele Valmasoni, Renato Salvador. Data curation: Matteo Santangelo, Luca Provenzano, Francesca Forattini, Matteo Pittacolo, Irene Sole Zuin, Loredana Nicoletti. Investigation: Luca Provenzano, Francesca Forattini, Matteo Pittacolo, Giulia Nezi, Loredana Nicoletti, Renato Salvador. Methodology: Arianna Vittori, Giovanni Capovilla, Rena Yadlapati, C. Prakash Gyawali, Edoardo Vincenzo Savarino. Data analysis and interpretation: Arianna Vittori, Giovanni Capovilla, Giulia Nezi. Writing – original draft: Arianna Vittori, Giovanni Capovilla, Matteo Santangelo. Writing – review and editing: All authors. Supervision: Edoardo Vincenzo Savarino, C. Prakash Gyawali, Rena Yadlapati, Michele Valmasoni, Renato Salvador. Final approval: All authors. Accountability: All authors.

## Funding

The authors have nothing to report.

## Disclosure

Renato Salvador received speaker and/or consulting fees from Aurora and Alfasigma; Edoardo Vincenzo Savarino has served as speaker for Abbvie, Aboca, Abivax, Agave, AGPharma, Alfasigma, Apoteca, Biosline, CaDiGroup, Celltrion, Dr. Falk, EG Stada Group, Eli Lilly, Fenix Pharma, Galapagos, Giuliani, Johnson&Johnson, JB Pharmaceuticals, Innovamedica/Adacyte, Lionhealth, Malesci, Mayoly Biohealth, Montefarco, Novartis, Omega Pharma, Pfizer, Rafa, Reckitt Benckiser, Recordati, Sandoz, Sanofi/Regeneron, SILA, Takeda, Tillots, Unifarco; has served as consultant for Abbvie, Alfasigma, Apogee, AstraZeneca, Biogen, Bristol‐Myers Squibb, Celltrion, Dr. Falk, Eli Lilly, Fenix Pharma, Ferring, Giuliani, Grunenthal, Johnson&Johnson, JB Pharmaceuticals, Merck & Co, Nestlè, Pfizer, PRO.MED.CS Praha a.s., Reckitt Benckiser, Recordati, Sanofi/Regeneron, SILA, Takeda, Unifarco; he received research support from Bonollo, Difass, Pfizer, Reckitt Benckiser, Sanofi/Regeneron, SILA, Unifarco, Zeta Farmaceutici; Rena Yadlapati has served as consultant for Phathom Pharmaceuticals, StatLinkMD, Braintree Pharmaceuticals, Reckitt Benckiser Healthcare Ltd., Medtronic; C. Prakash Gyawali has served as consultant for Medtronic, Phathom Pahramceuticals, Braintree Pharmaceuticals, Anaptys bio, Alimentiv, and has served as speaker and teacher for Carnot Pharmaceuticals. The remaining authors report no conflicts of interest relevant to this manuscript. The abstract was presented at Digestive Disease Week (DDW), Chicago, in May 2026.

## Conflicts of Interest

The authors declare no conflicts of interest.

## Supporting information


**Table S1:** STARD 2015 checklist for reporting diagnostic accuracy studies.
**Table S2:** STROBE statement—checklist of items that should be included in reports of cohort studies.
**Figure S1:** Flowchart of the study population. Created in BioRender.com (University of Padova license). HRM, high‐resolution manometry; LF, laparoscopic fundoplication.
**Table S3:** Demographic and preoperative clinical characteristics in the three groups: functioning and effective laparoscopic fundoplication (FELF), ineffective neo‐barrier (INB), and post‐fundoplication outflow obstruction (PFOO). AET, acid exposure time; BMI, body mass index; DCI, distal contractile integral; GERDQ, gastroesophageal reflux disease questionnaire; IRP, integrated relaxation pressure; LA, Los Angeles; LES, lower esophageal sphincter.
**Table S4:** Availability of each component of the composite reference standard, overall and according to the three Padova Classification diagnostic categories. Functioning and effective laparoscopic fundoplication (FELF); ineffective neo‐barrier (INB); post‐fundoplication outflow obstruction (PFOO).
**Table S5:** Cross‐tabulation of the index test (i.e., Padova Classification of post‐fundoplication high‐resolution manometry) versus reference standard for each investigated Padova Classification category.

## Data Availability

The data that support the findings of this study are available on request from the corresponding author. The data are not publicly available due to privacy or ethical restrictions.

## References

[1] B. Dallemagne , J. M. Weerts , C. Jehaes , S. Markiewicz , and R. Lombard , “Laparoscopic Nissen Fundoplication: Preliminary Report,” Surgical Laparoscopy & Endoscopy 1 (1991): 138–143. 1669393

[2] B. Dallemagne , J. Weerts , S. Markiewicz , et al., “Clinical Results of Laparoscopic Fundoplication at Ten Years After Surgery,” Surgical Endoscopy 20, no. 1 (2006): 159–165, 10.1007/s00464-005-0174-x. 16333553

[3] A. Simorov , A. Ranade , R. Jones , et al., “Long‐Term Patient Outcomes After Laparoscopic Anti‐Reflux Procedures,” Journal of Gastrointestinal Surgery 18, no. 1 (2014): 157–162, 10.1007/s11605-013-2401-4. 24234243

[4] J. J. Kelly , D. I. Watson , K. F. Chin , P. G. Devitt , P. A. Game , and G. G. Jamieson , “Laparoscopic Nissen Fundoplication: Clinical Outcomes at 10 Years,” Journal of the American College of Surgeons 205, no. 4 (2007): 570–575, 10.1016/j.jamcollsurg.2007.05.024. 17903731

[5] B. Robinson , C. M. Dunst , M. A. Cassera , K. M. Reavis , A. Sharata , and L. L. Swanstrom , “20 Years Later: Laparoscopic Fundoplication Durability,” Surgical Endoscopy 29, no. 9 (2015): 2520–2524, 10.1007/s00464-014-4012-x. 25487547

[6] R. Salvador , A. Vittori , G. Capovilla , et al., “Antireflux Surgery's Lifespan: 20 Years After Laparoscopic Fundoplication,” Journal of Gastrointestinal Surgery 27, no. 11 (2023): 2325–2335, 10.1007/s11605-023-05797-4. PMC10661768 37580489

[7] N. J. Soper and D. Dunnegan , “Anatomic Fundoplication Failure After Laparoscopic Antireflux Surgery,” Annals of Surgery 229, no. 5 (1999): 669, 10.1097/00000658-199905000-00009. PMC1420811 10235525

[8] R. Yadlapati , E. S. Hungness , and J. E. Pandolfino , “Complications of Antireflux Surgery,” American Journal of Gastroenterology 113, no. 8 (2018): 1137–1147, 10.1038/s41395-018-0115-7. PMC6394217 29899438

[9] R. Salvador , G. Capovilla , M. Santangelo , et al., “Manometric Identikit of a Functioning and Effective Fundoplication for Gastroesophageal Reflux Disease in the High‐Resolution Manometry ERA,” United European Gastroenterology Journal 12 (2024): 749–760. 10.1002/ueg2.12553 PMC11249834 38409901

[10] A. Vittori , G. Capovilla , R. Salvador , et al., “Laparoscopic Fundoplication Improves Esophageal Motility in Patients With Gastroesophageal Reflux Disease: A High‐Volume Single‐Center Controlled Study in the Era of High‐Resolution Manometry and 24‐Hour pH Impedance,” Journal of Gastrointestinal Surgery 29, no. 2 (2025): 101888, 10.1016/j.gassur.2024.101888. 39542415

[11] R. Salvador , J. E. Pandolfino , M. Costantini , et al., “The Role of High‐Resolution Manometry Prior to and Following Antireflux Surgery: The Padova Consensus,” Annals of Surgery 281 (2024): 124–135, 10.1097/SLA.0000000000006297. PMC11470131 38606560

[12] P. M. Bossuyt , J. B. Reitsma , D. E. Bruns , et al., “STARD 2015: An Updated List of Essential Items for Reporting Diagnostic Accuracy Studies,” BMJ 351 (2015): h5527, 10.1136/bmj.h5527. PMC4623764 26511519

[13] J. P. Vandenbroucke , E. von Elm , D. G. Altman , et al., “Strengthening the Reporting of Observational Studies in Epidemiology (STROBE): Explanation and Elaboration,” Epidemiology 18, no. 6 (2007): 805–835, 10.1097/EDE.0b013e3181577511. 18049195

[14] R. Jones , O. Junghard , J. Dent , et al., “Development of the GerdQ, a Tool for the Diagnosis and Management of Gastro‐Oesophageal Reflux Disease in Primary Care,” Alimentary Pharmacology & Therapeutics 30, no. 10 (2009): 1030–1038, 10.1111/j.1365-2036.2009.04142.x. 19737151

[15] M. Schroeder , A. Richardson , N. Shah , et al., “Validation of Dominant Symptom Intensity as a Patient‐Reported Outcome Measure in the Evaluation of Esophageal Symptoms,” Journal of Clinical Gastroenterology 60 (2026): 499–506. 10.1097/MCG.0000000000002352 41770658

[16] F. Forattini , K. H. N. Le , L. Provenzano , et al., “Validation of Padova Classification of Post‐Fundoplication Outflow Obstruction on High‐Resolution Manometry in an International Multi‐Center Study,” Neurogastroenterology and Motility 37 (2025): e70196. 10.1111/nmo.70196 PMC12623295 41184989

[17] P. O. Katz , L. B. Gerson , and M. F. Vela , “Guidelines for the Diagnosis and Management of Gastroesophageal Reflux Disease,” American Journal of Gastroenterology 108, no. 3 (2013): 308–328, 10.1038/ajg.2012.444. 23419381

[18] C. P. Gyawali , P. J. Kahrilas , E. Savarino , et al., “Modern Diagnosis of GERD: The Lyon Consensus,” Gut 67, no. 7 (2018): 1351–1362, 10.1136/gutjnl-2017-314722. PMC6031267 29437910

[19] C. P. Gyawali , R. Yadlapati , R. Fass , et al., “Updates to the Modern Diagnosis of GERD: Lyon Consensus 2.0,” Gut 73, no. 2 (2024): 361–371, 10.1136/gutjnl-2023-330616. PMC10846564 37734911

[20] J. M. de Oliveira , S. Birgisson , C. Doinoff , et al., “Timed Barium Swallow: A Simple Technique for Evaluating Esophageal Emptying in Patients With Achalasia,” AJR. American Journal of Roentgenology 169, no. 2 (1997): 473–479, 10.2214/ajr.169.2.9242756. 9242756

[21] S. W. Gan , N. Lee , S. E. Tan , S. M. Edwards , G. K. Kiroff , and J. C. Myers , “Quantification of Fluoroscopic Fundoplication Anatomy: Inter‐ and Intraobserver Reliability,” Diseases of the Esophagus 35, no. 2 (2022): doab045, 10.1093/dote/doab045. 34215875

[22] A. I. Carbo , R. H. Kim , T. Gates , and H. R. D'Agostino , “Imaging Findings of Successful and Failed Fundoplication,” Radiographics 34, no. 7 (2014): 1873–1884, 10.1148/rg.347130104. 25384289

[23] E. Savarino , N. de Bortoli , M. Bellini , et al., “Practice Guidelines on the Use of Esophageal Manometry ‐ A GISMAD‐SIGE‐AIGO Medical Position Statement,” Digestive and Liver Disease 48, no. 10 (2016): 1124–1135, 10.1016/j.dld.2016.06.021. 27443492

[24] A. Latorre‐Rodríguez , S. Mittal , and T. Masuda , A Complete Guide for Analyzing High‐Resolution Manometry Based on Chicago Classification Version 4.0 (Independently published, 2023).

[25] A. Juhasz , A. Sundaram , M. Hoshino , T. H. Lee , C. J. Filipi , and S. K. Mittal , “Endoscopic Assessment of Failed Fundoplication: A Case for Standardization,” Surgical Endoscopy 25, no. 12 (2011): 3761–3766, 10.1007/s00464-011-1785-z. 21643878

[26] L. R. Lundell , J. Dent , J. R. Bennett , et al., “Endoscopic Assessment of Oesophagitis: Clinical and Functional Correlates and Further Validation of the Los Angeles Classification,” Gut 45, no. 2 (1999): 172–180, 10.1136/gut.45.2.172. PMC1727604 10403727

[27] G. Zaninotto , P. Parente , R. Salvador , et al., “Long‐Term Follow‐Up of Barrett's Epithelium: Medical Versus Antireflux Surgical Therapy,” Journal of Gastrointestinal Surgery 16, no. 1 (2012): 7–14, 10.1007/s11605-011-1739-8. 22086718

[28] K. L. Lynch , C. P. Gyawali , E. Coss‐Adame , et al., “The Conundrum of Esophagogastric Junction Outflow Obstruction: Answers to Key Clinical Questions,” Annals of the New York Academy of Sciences 1549, no. 1 (2025): 5–10, 10.1111/nyas.15362. PMC12309437 40452117

[29] I. Le Blanc‐Louvry , E. Köning , A. Zalar , et al., “Severe Dysphagia After Laparoscopic Fundoplication: Usefulness of Barium Meal Examination to Identify Causes Other Than Tight Fundoplication–a Prospective Study,” Surgery 128, no. 3 (2000): 392–398, 10.1067/msy.2000.107981. 10965309

[30] J. G. Hunter , C. D. Smith , G. D. Branum , et al., “Laparoscopic Fundoplication Failures,” Annals of Surgery 230, no. 4 (1999): 595, 10.1097/00000658-199910000-00015. PMC1420908 10522729

[31] C. P. Dunn , S. E. Eriksson , B. A. Jobe , and S. Ayazi , “Endoscopy and Antireflux Surgery: A Technical Review of Pre‐ and Postoperative Evaluation and Recognizing Patterns of Failure,” Foregut 3, no. 4 (2023): 482–492, 10.1177/26345161231174246.

[32] T. H. Taft , J. R. Triggs , D. A. Carlson , et al., “Validation of the Oesophageal Hypervigilance and Anxiety Scale for Chronic Oesophageal Disease,” Alimentary Pharmacology & Therapeutics 47, no. 9 (2018): 1270–1277, 10.1111/apt.14605. PMC5897170 29528128

[33] T. H. Taft , D. A. Carlson , M. Simons , et al., “Esophageal Hypervigilance and Symptom‐Specific Anxiety in Patients With Eosinophilic Esophagitis,” Gastroenterology 161, no. 4 (2021): 1133–1144, 10.1053/j.gastro.2021.06.023. PMC8463417 34153298

[34] R. P. Tatum , R. V. Soares , E. Figueredo , B. K. Oelschlager , and C. A. Pellegrini , “High‐Resolution Manometry in Evaluation of Factors Responsible for Fundoplication Failure,” Journal of the American College of Surgeons 210, no. 5 (2010): 611–617, 10.1016/j.jamcollsurg.2009.12.023. 20421015

[35] M. Hoshino , A. Srinivasan , and S. K. Mittal , “High‐Resolution Manometry Patterns of Lower Esophageal Sphincter Complex in Symptomatic Post‐Fundoplication Patients,” Journal of Gastrointestinal Surgery 16, no. 4 (2012): 705–714, 10.1007/s11605-011-1803-4. 22231632

[36] D. T. Müller , B. Parker , R. Fletcher , et al., “High Resolution Manometry in a Functioning Fundoplication ‐ Establishing a Standard Profile: Retrospective Chart Review,” Annals of Surgery 276, no. 6 (2022): e764–e769, 10.1097/SLA.0000000000004813. 33630441

[37] P. W. Weijenborg , E. Savarino , B. F. Kessing , et al., “Normal Values of Esophageal Motility After Antireflux Surgery; a Study Using High‐Resolution Manometry,” Neurogastroenterology and Motility 27, no. 7 (2015): 929–935, 10.1111/nmo.12554. 26095116

[38] J. A. Broeders , W. A. Draaisma , D. R. de Vries , A. J. Bredenoord , A. J. Smout , and H. G. Gooszen , “The Preoperative Reflux Pattern as Prognostic Indicator for Long‐Term Outcome After Nissen Fundoplication,” American Journal of Gastroenterology 104, no. 8 (2009): 1922–1930, 10.1038/ajg.2009.228. 19491839

[39] J. Miholic , J. Hafez , J. Lenglinger , et al., “Hiatal Hernia, Barrett's Esophagus, and Long‐Term Symptom Control After Laparoscopic Fundoplication for Gastroesophageal Reflux,” Surgical Endoscopy 26, no. 11 (2012): 3225–3231, 10.1007/s00464-012-2328-y. 22648102

[40] P. R. Armijo , B. Pokala , M. Misfeldt , S. Pagkratis , and D. Oleynikov , “Predictors of Hiatal Hernia Recurrence After Laparoscopic Anti‐Reflux Surgery With Hiatal Hernia Repair: A Prospective Database Analysis,” Journal of Gastrointestinal Surgery 23, no. 4 (2019): 696–701, 10.1007/s11605-018-04073-0. 30617774

[41] S. Kapadia , T. Osler , A. Lee , and E. Borrazzo , “The Role of Preoperative High Resolution Manometry in Predicting Dysphagia After Laparoscopic Nissen Fundoplication,” Surgical Endoscopy 32, no. 5 (2018): 2365–2372, 10.1007/s00464-017-5932-z. 29234939

[42] H. Al Asadi , H. Najah , Y. Li , et al., “Determination of Causes of Post‐Operative Dysphagia After Anti‐Reflux Surgery Based on Intra‐Operative Planimetry,” Surgical Endoscopy 38, no. 10 (2024): 5623–5633, 10.1007/s00464-024-11101-5. 39101988

[43] O. Riedl , M. Gadenstätter , W. Lechner , G. Schwab , M. Marker , and R. Ciovica , “Preoperative Lower Esophageal Sphincter Manometry Data Neither Impact Manifestations of GERD nor Outcome After Laparoscopic Nissen Fundoplication,” Journal of Gastrointestinal Surgery 13, no. 7 (2009): 1189–1197, 10.1007/s11605-009-0890-y. 19370381

