# Supplementary material for: Diagnostic Accuracy of High‐Resolution Manometry for Assessing Fundoplication Integrity: Validation of the Padova Classification
Source: Neurogastroenterol Motil. 2026 Oct 2;38(10):e70457. doi: 10.1111/nmo.70457 (PMC13631549; doi:10.1111/nmo.70457)
Supplement: Supplementary file 1 [file NMO-38-e70457-s001.docx]

**Supplementary Table 1.** STARD 2015 checklist for reporting diagnostic accuracy studies

|  | **Section & Topic** | **No** | **Item** | **Reported on page #** |
| --- | --- | --- | --- | --- |
|  |  |  |  |  |
|  | **TITLE OR ABSTRACT** |  |  |  |
|  |  | **1** | Identification as a study of diagnostic accluracy using at least one measure of accuracy (such as sensitivity, specificity, predictive values, or AUC) | 1 |
|  | **ABSTRACT** |  |  |  |
|  |  | **2** | Structured summary of study design, methods, results, and conclusions  (for specific guidance, see STARD for Abstracts) | 3-4 |
|  | **INTRODUCTION** |  |  |  |
|  |  | **3** | Scientific and clinical background, including the intended use and clinical role of the index test | 5 |
|  |  | **4** | Study objectives and hypotheses | 5-6 |
|  | **METHODS** |  |  |  |
|  | *Study design* | **5** | Whether data collection was planned before the index test and reference standard  were performed (prospective study) or after (retrospective study) | 6 |
|  | *Participants* | **6** | Eligibility criteria | 6 |
|  |  | **7** | On what basis potentially eligible participants were identified  (such as symptoms, results from previous tests, inclusion in registry) | 6 |
|  |  | **8** | Where and when potentially eligible participants were identified (setting, location and dates) | 6 |
|  |  | **9** | Whether participants formed a consecutive, random or convenience series | 11 |
|  | *Test methods* | **10a** | Index test, in sufficient detail to allow replication | 7 |
|  |  | **10b** | Reference standard, in sufficient detail to allow replication | 7-8 |
|  |  | **11** | Rationale for choosing the reference standard (if alternatives exist) | 9-10 |
|  |  | **12a** | Definition of and rationale for test positivity cut-offs or result categories  of the index test, distinguishing pre-specified from exploratory | 8-9 |
|  |  | **12b** | Definition of and rationale for test positivity cut-offs or result categories  of the reference standard, distinguishing pre-specified from exploratory | 9-10 |
|  |  | **13a** | Whether clinical information and reference standard results were available  to the performers/readers of the index test | 9 |
|  |  | **13b** | Whether clinical information and index test results were available  to the assessors of the reference standard | 10 |
|  | *Analysis* | **14** | Methods for estimating or comparing measures of diagnostic accuracy | 11 |
|  |  | **15** | How indeterminate index test or reference standard results were handled | 9,10 |
|  |  | **16** | How missing data on the index test and reference standard were handled | 6 |
|  |  | **17** | Any analyses of variability in diagnostic accuracy, distinguishing pre-specified from exploratory | 11 |
|  |  | **18** | Intended sample size and how it was determined | 11 |
|  | **RESULTS** |  |  |  |
|  | *Participants* | **19** | Flow of participants, using a diagram | Figure S3 |
|  |  | **20** | Baseline demographic and clinical characteristics of participants | Table S4 |
|  |  | **21a** | Distribution of severity of disease in those with the target condition | NA |
|  |  | **21b** | Distribution of alternative diagnoses in those without the target condition | NA |
|  |  | **22** | Time interval and any clinical interventions between index test and reference standard | NA |
|  | *Test results* | **23** | Cross tabulation of the index test results (or their distribution)  by the results of the reference standard | Table S5 |
|  |  | **24** | Estimates of diagnostic accuracy and their precision (such as 95% confidence intervals) | 14 |
|  |  | **25** | Any adverse events from performing the index test or the reference standard | NA |
|  | **DISCUSSION** |  |  |  |
|  |  | **26** | Study limitations, including sources of potential bias, statistical uncertainty, and generalisability | 19 |
|  |  | **27** | Implications for practice, including the intended use and clinical role of the index test | 19 |
|  | **OTHER INFORMATION** |  |  |  |
|  |  | **28** | Registration number and name of registry | NA |
|  |  | **29** | Where the full study protocol can be accessed | NA |
|  |  | **30** | Sources of funding and other support; role of funders | NA |
|  |  |  |  |  |

**Supplementary Table 2.** STROBE Statement – Checklist of items that should be included in reports of cohort studies

|  | | **Item No** | **Recommendation** | **Page No** | |
| --- | --- | --- | --- | --- | --- |
| **Title and abstract** | | 1 | (*a*) Indicate the study’s design with a commonly used term in the title or the abstract |  | |
|  |  |  | (*b*) Provide in the abstract an informative and balanced summary of what was done and what was found | 1-4 | |
| **Introduction** | | | | | |
| Background/rationale | | 2 | Explain the scientific background and rationale for the investigation being reported | 5 | |
| Objectives | | 3 | State specific objectives, including any prespecified hypotheses | 5-6 | |
| **Methods** | | | | | |
| Study design | | 4 | Present key elements of study design early in the paper | 6 | |
| Setting | | 5 | Describe the setting, locations, and relevant dates, including periods of recruitment, exposure, follow-up, and data collection | 6 | |
| Participants | | 6 | (*a*) Give the eligibility criteria, and the sources and methods of selection of participants. Describe methods of follow-up | 6-10 | |
|  |  |  | (*b*) For matched studies, give matching criteria and number of exposed and unexposed |  | |
| Variables | | 7 | Clearly define all outcomes, exposures, predictors, potential confounders, and effect modifiers. Give diagnostic criteria, if applicable | 6-10 | |
| Data sources/ measurement | | 8* | For each variable of interest, give sources of data and details of methods of assessment (measurement). Describe comparability of assessment methods if there is more than one group | 6-10 | |
| Bias | | 9 | Describe any efforts to address potential sources of bias | 9-10 | |
| Study size | | 10 | Explain how the study size was arrived at | 11 | |
| Quantitative variables | | 11 | Explain how quantitative variables were handled in the analyses. If applicable, describe which groupings were chosen and why | 11 | |
| Statistical methods | | 12 | (*a*) Describe all statistical methods, including those used to control for confounding | 11 | |
|  |  |  | (*b*) Describe any methods used to examine subgroups and interactions |  | |
|  |  |  | (*c*) Explain how missing data were addressed |  | |
|  |  |  | (*d*) If applicable, explain how loss to follow-up was addressed |  | |
|  |  |  | (*e*) Describe any sensitivity analyses |  | |
| **Results** | | | |  | |
| Participants | | 13* | (a) Report numbers of individuals at each stage of study—eg numbers potentially eligible, examined for eligibility, confirmed eligible, included in the study, completing follow-up, and analysed | Figure S3 | |
|  |  |  | (b) Give reasons for non-participation at each stage |  | |
|  |  |  | (c) Consider use of a flow diagram |  | |
| Descriptive data | | 14* | (a) Give characteristics of study participants (eg demographic, clinical, social) and information on exposures and potential confounders | Table S4 | |
|  |  |  | (b) Indicate number of participants with missing data for each variable of interest |  | |
|  |  |  | (c) Summarise follow-up time (eg, average and total amount) |  | |
| Outcome data | | 15* | Report numbers of outcome events or summary measures over time | NA | |
| Main results | 16 | | (*a*) Give unadjusted estimates and, if applicable, confounder-adjusted estimates and their precision (eg, 95% confidence interval). Make clear which confounders were adjusted for and why they were included | | 12-14 |
|  |  |  | (*b*) Report category boundaries when continuous variables were categorized | |  |
|  |  |  | (*c*) If relevant, consider translating estimates of relative risk into absolute risk for a meaningful time period | |  |
| Other analyses | 17 | | Report other analyses done—eg analyses of subgroups and interactions, and sensitivity analyses | | 12-14 |
| **Discussion** | | | | | |
| Key results | 18 | | Summarise key results with reference to study objectives | | 15 |
| Limitations | 19 | | Discuss limitations of the study, taking into account sources of potential bias or imprecision. Discuss both direction and magnitude of any potential bias | | 19 |
| Interpretation | 20 | | Give a cautious overall interpretation of results considering objectives, limitations, multiplicity of analyses, results from similar studies, and other relevant evidence | | 19 |
| Generalisability | 21 | | Discuss the generalisability (external validity) of the study results | | 15-19 |
| **Other information** | | | | | |
| Funding | 22 | | Give the source of funding and the role of the funders for the present study and, if applicable, for the original study on which the present article is based | | NA |

**Supplementary Figure 1.** Flowchart of the study population. Created in [https://BioRender.com](https://biorender.com/) (University of Padova license). HRM, high-resolution manometry; LF, laparoscopic fundoplication

**Supplementary Table 3.** Demographic and preoperative clinical characteristics in the three groups: functioning and effective laparoscopic fundoplication (FELF), ineffective neo-barrier (INB), and post-fundoplication outflow obstruction (PFOO). AET, acid exposure time; BMI, body mass index; DCI, distal contractile integral; GERDQ, gastroesophageal reflux disease questionnaire; IRP, integrated relaxation pressure; LA, Los Angeles; LES, lower esophageal sphincter.

| **Characteristic** | **FELF**   N = 219^1^ | **INB**   N = 80^1^ | **PFOO**   N = 67^1^ | **p-value**^2^ |
| --- | --- | --- | --- | --- |
| **Age, years** | 53 (42–63) | 54 (45–64) | 56 (44–66) | 0.5 |
| **Sex** |  |  |  | 0.5 |
| Female | 96 (44%) | 30 (38%) | 31 (46%) |  |
| Male | 123 (56%) | 50 (63%) | 36 (54%) |  |
| **BMI, kg/m^2^** | 25.6 (23.9–28.1) | 26.9 (23.5–27.9) | 26.5 (22.2–29.4) | 0.7 |
| **GERDQ score** | 10 (8–16) | 12 (9–16) | 11 (8–16) | 0.9 |
| **LES total length, mm** | 24 (19–32) | 22 (16–29) | 30 (26–41) | **<0.001** |
| **LES abdominal length, mm** | 0 (0–13) | 0 (0–9) | 10 (0–18) | **0.003** |
| **LES basal pressure, mmHg** | 16 (9–24) | 10 (4–19) | 21 (10–27) | **0.004** |
| **IRP, mmHg** | 5.2 (2.5–8.9) | 3.6 (1.0–6.0) | 5.7 (1.0–11.9) | **0.042** |
| **DCI, mmHg·s·cm** | 599 (351–761) | 398 (215–628) | 477 (261–770) | 0.075 |
| **AET, %** | 9 (6–13) | 15 (8–23) | 9 (6–15) | **0.001** |
| **Esophagitis, LA grade**^3^ |  |  |  | 0.21 |
| No esophagitis | 97 (56%) | 29 (44%) | 22 (51%) |  |
| A | 18 (10%) | 8 (12%) | 5 (12%) |  |
| B | 30 (17%) | 8 (12%) | 5 (12%) |  |
| C | 22 (13%) | 9 (14%) | 5 (12%) |  |
| D | 7 (4.0%) | 9 (14%) | 6 (14%) |  |
| **Barrett’s esophagus** | 0 (0%) | 3 (4.5%) | 0 (0%) | **0.007** |
| **Fundoplication type** |  |  |  | **<0.001** |
| Nissen | 100 (46%) | 55 (69%) | 50 (75%) |  |
| Toupet | 119 (54%) | 25 (31%) | 17 (25%) |  |

^1^Median (Q1–Q3); n (%)

^2^Kruskal-Wallis rank sum test; Pearson's Chi-squared test or Fisher’s exact test, as appropriate

^3^Percentages based on available endoscopic data

**Supplementary Table 4.** Availability of each component of the composite reference standard, overall and according to the three Padova classification diagnostic categories. Functioning and effective laparoscopic fundoplication (FELF); ineffective neo-barrier (INB); post-fundoplication outflow obstruction (PFOO)

| **Component of composite reference standard** | **Overall**  N = 366* | **FELF**  N = 219 | **INB**  N = 80 | **PFOO**  N = 67 |
| --- | --- | --- | --- | --- |
| Clinical assessment available | 366 (100%) | 219 (100%) | 80 (100%) | 67 (100%) |
| Barium swallow available | 351 (95.9%) | 207 (94.5%) | 79 (98.8%) | 65 (97%) |
| Ambulatory pH monitoring available | 341 (93.1%) | 201 (91.7%) | 77 (96.3%) | 63 (94%) |
| Upper endoscopy available | 325 (88.7%) | 191 (87.2%) | 74 (92.5%) | 60 (89.6%) |
| All three investigations available | 291 (79.5%) | 167 (76.3%) | 70 (87.5%) | 54 (80.6%) |

* Among the 373 patients included in the study cohort, seven were diagnosed with absent contractility and were therefore excluded from further statistical analyses.

**Supplementary Table 5.** Cross-tabulation of index test (i.e., Padova Classification of post-fundoplication high resolution manometry) vs reference standard for each investigated Padova classification category

|  |  | Reference Standard | |
| --- | --- | --- | --- |
|  |  | FELF | Other diagnoses |
| Padova Classification | FELF | 215 | 4 |
|  | Other diagnoses | 12 | 142 |
|  |  |  |  |
|  |  | Reference Standard | |
|  |  | PFOO | Other diagnoses |
| Padova Classification | PFOO | 59 | 8 |
|  | Other diagnoses | 0 | 306 |
|  |  |  |  |
|  |  | Reference Standard | |
|  |  | INB | Other diagnoses |
| Padova Classification | INB | 71 | 9 |
|  | Other diagnoses | 9 | 284 |
